# Astrocytes and Microglia in Stress-Induced Neuroinflammation: The African Perspective

**DOI:** 10.3389/fimmu.2022.795089

**Published:** 2022-05-30

**Authors:** Matthew Ayokunle Olude, Abdeslam Mouihate, Oluwaseun Ahmed Mustapha, Cinthia Farina, Francisco Javier Quintana, James Olukayode Olopade

**Affiliations:** ^1^ Vertebrate Morphology, Environmental Toxicology and Neuroscience Unit, College of Veterinary Medicine, Federal University of Agriculture, Abeokuta, Nigeria; ^2^ Department of Physiology, Faculty of Medicine, Health Sciences Centre, Kuwait University, Kuwait City, Kuwait; ^3^ Istituto di Ricovero e Cura a Carattere Scientifico (IRCSS) San Raffaele Scientific Institute, Institute of Experimental Neurology (INSPE) and Division of Neuroscience, Milan, Italy; ^4^ Ann Romney Center for Neurologic Diseases, Brigham and Women’s Hospital, Harvard Medical School, Boston, MA, United States; ^5^ Neuroscience Unit, Department of Veterinary Anatomy, Faculty of Veterinary Medicine, University of Ibadan, Ibadan, Nigeria

**Keywords:** astrocytes, microglia, reactive oxygen species, malnutrition, environmental pollution

## Abstract

**Background:** Africa is laden with a youthful population, vast mineral resources and rich fauna. However, decades of unfortunate historical, sociocultural and leadership challenges make the continent a hotspot for poverty, indoor and outdoor pollutants with attendant stress factors such as violence, malnutrition, infectious outbreaks and psychological perturbations. The burden of these stressors initiate neuroinflammatory responses but the pattern and mechanisms of glial activation in these scenarios are yet to be properly elucidated. Africa is therefore most vulnerable to neurological stressors when placed against a backdrop of demographics that favor explosive childbearing, a vast population of unemployed youths making up a projected 42% of global youth population by 2030, repressive sociocultural policies towards women, poor access to healthcare, malnutrition, rapid urbanization, climate change and pollution. Early life stress, whether physical or psychological, induces neuroinflammatory response in developing nervous system and consequently leads to the emergence of mental health problems during adulthood. Brain inflammatory response is driven largely by inflammatory mediators released by glial cells; namely astrocytes and microglia. These inflammatory mediators alter the developmental trajectory of fetal and neonatal brain and results in long-lasting maladaptive behaviors and cognitive deficits. This review seeks to highlight the patterns and mechanisms of stressors such as poverty, developmental stress, environmental pollutions as well as malnutrition stress on astrocytes and microglia in neuroinflammation within the African context.

## Introduction

### Astrocytes: From Physiology to Neuroinflammation

Astrocytes are glial cells of the central nervous system (CNS) of neuroectodermal origin. In fact, neurons, oligodendrocytes and astrocytes derive from a common multipotent self-renewable neural stem cell in a process that occurs with precise timing (1). While neurogenesis takes place early during embryonic development and is accomplished at about birth, gliogenesis follows neurogenesis and is finalized in postnatal life (1), with synaptogenesis and neuronal function depending on astrocyte morphology, maturation and regional specification (1).

Astrocytes play key physiological functions in the CNS that, if altered, may lead to or amplify tissue damage and neuroinflammation and hamper relevant brain functions, such as cognition and memory.

First, astrocytes contribute to the complexity of CNS structure. Evolution has led to the relative expansion of astrocytes, especially in the human brain where the number of astrocytes exceeds that of neurons and astrocytes present complex arborisation architectures (2). CNS damage triggers a complex response in astrocytes, which become reactive and undergo transcriptional remodelling, early upregulation of the intermediate filament glial fibrillary acidic protein (GFAP), morphological changes and proliferation (3). This reaction alters physiological tissue topology and may lead to scar formation as observed in traumatic, neuroinflammatory and neurodegenerative CNS disorders (3, 4). By contrast, decreased astrocyte numbers and GFAP signals have been evidenced in mental disorders (5), underlying a distinct pattern of astrocyte pathology. Astrocytes are involved in the control of neuroinflammation, as conventional or inducible GFAP deficient mice display exacerbated expression of Toxoplasma encephalitis, Staphylococcus aureus-induced brain abscess, spinal cord and brain injury (reviewed in 6). In the experimental model of multiple sclerosis, the experimental autoimmune encephalomyelitis (EAE), astrocyte depletion may worsen or attenuate disease depending on whether depletion occurs immediately after EAE induction or during the chronic phase respectively (7, 8), indicating that GFAP positive cells may display protective or detrimental functions at distinct stages of disease.

Second, astrocytes regulate neuronal survival *via* release of and/or response to crucial mediators like neurotrophins, well known growth factors for neurons to which astrocytes may become sensitive under pathological conditions and react with the synthesis of neurotoxic nitric oxide (9, 10). Furthermore, astrocytes sense neuronal activity as their fine extensions take contact with pre- and post-synaptic neurons (forming the so-called tripartite synapse) and bear neurotransmitter receptors (11). They may modulate the concentration of glutamate, the main excitatory neurotransmitter in the brain, present in the synaptic cleft *via* specific transporters called glutamate/aspartate transporter (GLAST) and Astrocytic Glutamate Transporter 1 (GLT1) (11). *In vitro* and *in vivo* evidences indicate that neuroinflammation is characterized by alterations in expression of glutamate transporters and glutamate buffering (12, 13). The accumulation of glutamate in the extracellular space causes neuronal damage by excitotoxicity, a phenomenon observed in neurological and psychiatric disorders (reviewed in 14). Furthermore, astrocytes can provide metabolic precursors of glutamate back to the neurons through monocarboxylate transporters (MCT), thus satisfying neuronal metabolic needs and limiting novel neurotransmitter synthesis (15, 16) Though scarce information is available about astrocyte metabolism in neuroinflammatory mouse models, it is known that respiration-deficient astrocytes may survive as glycolytic cells *in vivo* in the absence of tissue inflammation and damage and that inflammatory cytokines increase glycolytic rates of astrocytes *in vitro* (17, 18), suggesting sustained glycolytic proficiency of the astrocyte in neuroinflammation. Interestingly, disruption of MCT transporters in astrocytes *in vivo* causes amnesia, underlying a key role for astrocyte-neuron metabolic coupling in long term memory formation (19).

Third, neuronal activity strongly depends on continuous supply of oxygen and glucose through the cerebral blood flow (15). Astrocytes cover most of the cerebral vasculature and create neurovascular units which link synaptic activity to vessel tone, thus regulating microcirculation (15). Further, astrocytes are key constituents of the blood-brain barrier (BBB) and their interaction with endothelial cells regulates BBB development and function (20). On the one hand, astrocyte factors control formation of tight junctions, blood flow, microvascular permeability, cell matrix and angiogenesis; on the other hand, endothelial signals regulate astrocyte maturation and expression of receptor proteins and ion channels on the glial membrane (20). Thus, for example Aquaporin-4 (AQP4) expression at astrocytic endfeet in contact with the vasculature together with the inward rectifying K+ channel Kir 4.1 provides local control of water and ion homeostasis (20). Autoantibodies directed to AQP4 are at the basis of the pathogenesis of neuromyelitis optica (NMO), an inflammatory CNS disorder characterized by astrocyte loss, axonal damage and demyelination (21–23), while the occurrence of anti-Kir 4.1 antibodies in MS is controversial (24). Further, Kir 4.1 has been reported as downregulated in ALS and epilepsy (25, 26), while upregulated in animal models of depression (27). Notably, mice lacking astrocyte Kir 4.1 display ataxia and seizures and die prematurely (28) while animals overexpressing astrocytic Kir4.1 develop a depression-like phenotype (27).

Fourth, astrocytes can exert and control immune reactions in the CNS (29–31). Similarly, to microglia, they bear a repertoire of pattern recognition receptors (PRR), which allow recognition of genome, proteins, and glycolipids of microbial origin (aka pathogen-associated molecular patterns, PAMP) (29). PRR include toll-like receptors, scavenger receptors and complement factors and have been initially identified as tools of the innate immune system to fight infections (29). However, PRR also recognize danger signals, that are endogenous molecules released or activated during stress or damage under sterile conditions (and collectively called DAMP, damage-associated molecular patterns) (32). DAMP include molecules from the extracellular matrix (e.g. biglycan and fibrinogen), cytosol (e.g. S100 proteins and heat shock proteins), and nucleus (e.g. histones) (reviewed in 32) Activation of innate immune pathways has been demonstrated in infectious, autoimmune, neurodegenerative disorders (29, for review). PRR engagement on glia cells activates pro-inflammatory responses required to eliminate or, at least, to contain infectious agents or damage. Critical is the activation of the transcription factor NF-kB, which controls gene expression of inflammatory cytokines, chemokines, nitric oxide synthase, apoptosis regulators (6, 29). In fact, *in vivo* inhibition of NFkB signalling in astrocytes protects from spinal cord and brain injury, EAE and toxic demyelination (33–36).

While rare in physiology, under pathological settings reactive astrocytes may modulate adaptive immunity in the CNS in several ways. Reactive astrocytes may release chemokines, such as CXCL10 and CCL2, which attract T cells from the circulation into the CNS parenchyma (37, 38) and *in vivo* deletion of astrocyte CCL2 and CXCL10 protects from EAE (39, 40). Next, T cell-derived factors such as IFN γ stimulate the expression of MHC class II molecules on astrocyte membrane, so that these glia cells become efficient in presentation of CNS antigens (e.g. myelin proteins) to T cells (41, 42), thus potentially sustaining local autoreactive adaptive immune responses. On the other hand, IFN γ can be released also by regulatory T cells and IFN γ signalling has been shown to be protective *in vivo* in EAE (43–45). Astrocytes are also great producers of TGFbeta, a known immunosuppressive mediator, and blockade of TGFbeta synthesis in astrocytes enhances tissue pathology in stroke and infectious CNS models (46). Further, astrocytes have been shown to express CTLA4, CD39 and CD73 (47, 48), which limit T cell activation, and FasL and TRAIL which trigger T cell deletion (31, 49). Regarding the interaction with B lymphocytes, astrocytes may release CXCL12, which promotes B cell recruitment to the CNS (50) and BAFF, a mediator important for B cell development, survival and function (51). Overall, these observations indicate a key role for astrocytes in regulating adaptive immune reactions. Activated astrocytes support B cell survival and activation, in turn, activated B cells induce a better T cell proliferation (52).

### Microglia in Health and Disease

Microglia, a set of small glial cells within the CNS, were first described by del Río Hortega (53). Several decades passed before the importance of microglial functions in the CNS were appreciated. In 1920s, del Río Hortega had provided histological evidence that these cells derive from the mesoderm and not the ectoderm; the source of all other neural cells (oligodendrocytes, neurons and astrocytes) (53–55). It is now accepted that these glial cells originate in the yolk sac during fetal development and emerge at an earlier stage than tissue macrophages (56–58). Under basal condition, microglia display a multitude of physiological effects in such cellular processes as neurogenesis, cerebral angiogenesis, synaptic pruning, and oligodendrogenesis during brain development in both rodents and primates (59–61). Indeed, microglia contribute to neurogenesis and olidendrogenesis during prenatal and neonatal period (59, 62, 63). They emerge concomitantly with newly-born neurons and heavily invade neurogenic niches such as the ventricular and subventricular zones. This spatiotemporal co-existence between microglia and newly-born neural cells (neurons and oligodendrocytes) indicate the potential role of microglia in the regulation of neurogenesis and oligodendrogenesis (62, 63). For example, a subgroup of microglia expressing CD11c play a major role in the initial phase of myelination in developing brain (61). Through their phagocytic activity, microglia contribute to the removal of cell debris of dying neural cells and create optimal environment for neuronal connectivity.

Microglia affect cell survival/death programs of neural cells and remodel synaptic connection between developing neurons by secreting a variety of pro- and anti-inflammatory cytokines (Tumor Necrosis Factor (TNFα), Interleukin 1β (IL-1β), IL-6 and IL-10) and growth factors such as insulin growth factor 1 (IGF1) and brain-derived neurotrophic factor (BDNF) (61, 64–67). Thus, any alteration to these developmental effects of microglial can have long-lasting impact on brain structure and function (65, 67–69).

In addition to their homeostatic effects, microglia play a major role in the immune response to a variety of insults including pathogens (viral, bacterial or parasitic), trauma, stroke, and neurodegenerative diseases (70). For their immune-related function, microglia are referred to as the immune-competent cells of the CNS. Under basal condition, microglia form a network of cells characterized with small perikarya and long thin processes. These processes dynamically “sniff” their environment for signs of tissue damage such as high extracellular concentrations of calcium ions or adenosine triphosphate (ATP) (71). A damage to neural tissues triggers a cascade of cellular events within microglia. These cells send long processes towards the site of damage and adopt morphological changes whereby their cell bodies become enlarged and adopt an amoeboid shape. Their processes become short and thick. The genetic program of activated microglia is shifted towards cell division and phagocytosis and is characterized by the synthesis and release of a myriad of inflammatory cytokines and trophic factors (71). These cellular and molecular responses appear to be beneficial during the acute phase of insult. However, a prolonged activation of microglia can become deleterious (72, 73). While microglial cellular response appears to be non-specific, many line of evidence suggest that these cells exert a strong pro-inflammatory response during the initial phase of insult followed later by a regulatory response that consist mainly in the production of anti-inflammatory cytokines (IL-4 and IL10) and trophic factors (73, 74). The secondary wave of anti-inflammatory cytokines and trophic factors contributes to the recovery process of CNS tissue from the injury.

### Astrocytes and Microglia Cross-Talk

Cell-cell interactions control CNS physiology and pathology (6, 75–84). Astrocyte-microglia interactions, for example, play important roles in CNS development, health and disease (31, 85, 86). In 2012 Ben Barres and colleagues reported that LPS induces a neurotoxic phenotype in astrocytes (87). Follow up mechanistic studies stablished that LPS induces the production of IL-1α, TNF-α, and complement component 1q (C1q) by microglia, which act on astrocytes to induce neurotoxic activity mediated by a lipid and additional as-yet unidentified neurotoxic factor (79, 88). In addition, these microglia-induced neurotoxic astrocytes display decreased phagocytic activity, and the reduced expression of neurotrophic factors (79). Finally, the analysis of patient samples suggest that these neurotoxic astrocytes contribute to the pathology of multiple neurologic diseases, including Huntington’s disease, Alzheimer’s disease, and multiple sclerosis (MS), among others (79). Collectively, these findings opened new avenues about the microglial regulation of astrocyte responses, and its contribution to CNS pathology. For instance, microglia can likely produce both positive and negative regulators of astrocyte pathogenic responses (see 80). Several molecules have been found to be involved in astrocyte-microglia communication, and the control of these cell-cell interactions by the commensal flora in specific diseases such as MS (89–96). For instance, VEGF-B was identified as a microglial product that boosts disease-promoting astrocyte responses. The transcription factor aryl hydrocarbon receptor (AHR) in microglia boosts TGFα while repressing the production of VEGF-B. Furthermore, AHR can also be activated in the CNS by metabolites produced by the commensal gut flora (which as a result of their chemical structure cross the BBB) and induced by environmental chemicals (83, 97–99). These thus contribute to regulation of astrocyte-microglia communication and CNS pathology (see 100).

Astrocytes can control microglia responses (100). Although multiple mechanisms likely mediate the control of microglial responses by astrocytes, some candidate pathways have already been identified. For example, fate-mapping and other studies established that astrocytes produce GM-CSF (100), 101), a known regulator of microglial activation (84, 102, 103). Astrocytes have been shown to modulate microglial responses *via* the production of GM-CSF (8, 104). Similar observations have been made for IL-6 (105–109). The above-mentioned findings exemplify the important role astrocyte-microglia interactions in CNS physiology. The recently developed RABID-seq (**Ra**bies **B**arcoding **I**n **D**roplets) which uses a library of genetically barcoded rabies virus in combination with single-cell RNA-seq to study CNS cell-cell interactions *in vivo* (110), identifying interacting cells, the mechanisms involved, and the biologic consequences of those interactions, has helped to highlight an important role for microglial-astrocyte interactions mediated by EphrinB3 and EphB3 in the promotion of CNS pathology (110) by inducing proinflammatory gene expression in the CNS (111), potentially *via* the activation of MAPK and the NLRP3 inflammasome (112, 113). In addition, EphB3 signaling in astrocytes induces the production of D-serine (114), which promotes synaptic damage *via* NMDA receptors (115). We also found that EphB3 in astrocytes is activated by its membrane-bound ligand EphrinB3 expressed by microglia. Interestingly, EphrinB3 harbors an intracellular domain that can trigger specific signaling pathways. Indeed, reverse signaling *via* EphrinB3 boosts the expression of NF-kB-driven transcriptional programs in microglia that promote inflammation and neurodegeneration (see 104, 110).

### Early-Life Immune Challenge

In Africa, vast populations are exposed to stressors across all age groups with early life exposures carrying the greatest neurological burdens. These early life challenges alter the developmental trajectory of the CNS and consequently result in neurodevelopmental disorders (116). Epidemiological studies have shown a correlation between early life immune challenge and brain related diseases such as schizophrenia (117, 118), autism spectrum disorder (119, 120) and attention deficit hyperactivity disorder (121). It was suggested that the emergence of these brain related diseases are linked to altered early life function of microglia, as these cells play a pivotal role in synaptic pruning, neuronal connectivity and removal of dying neurons during brain development (122). Furthermore, depletion of microglia during early life induces persistent changes in social behavior such as reduced anxiety-like behavior and impaired working memory (123, 124). These effects were absent when microglial activity was inhibited during adulthood (125).

Experimental studies have shown that early life exposure to pathogens such as bacteria or viruses alters brain development trajectory and consequently leads to persistent cognitive deficits and behavioral dysfunctions. Indeed prenatal or neonatal exposures to either viral mimetics (polyinosinic:polycytidylic acid: PolyI:C) or bacterial active ingredient (Lipopolysaccharide: LPS) reprograms the hypothalamic-pituitary adrenal axis and affects brain development and plasticity that lasts into adulthood (126–129). These long-lasting effects are not due to the pathogens *per se*, but are triggered by maternal immune response to these pathogens (130, 131). We and others have shown that maternal immune activation alters adult brain plasticity and cognitive functions *via* maternally borne mediators such as interleukin-6 (IL-6) (132–134) and transforming growth factor-β (TGF-β) (135).

In addition to pathogens, non-infectious agents such as stress (136–138) or exposure to air pollution (diesel exhaust particles) can also activate maternal immune system and consequently alters fetal brain development (139). Indeed, exposure to these non-infectious agents induces microgliosis in the fetal brain and leads to an enhanced reactivity of microglia later in life, which is accompanied with cognitive dysfunction such as learning and memory deficits (140).

### Long-Lasting Impact of Maternal Infection in Africa

The major cause of deaths in sub-Saharan Africa is infectious diseases (69%). A significant percentage of these deaths is associated with infection during pregnancy because pregnancy is characterized by an immune tolerant state to prevent rejection of the fetus (141–143). A relatively large epidemiological study has shown that the frequency of maternal infection and its resulting complication was higher in African low-income countries (15 African countries), when compared to high-income non-African countries. Indeed, obstetric infection led to maternal mortality of about 10.7% in low-income countries when compared to that seen in high income countries (about 4.7%). These infections include urinary tract infections, chorioamnionitis and abortion related infections (144, 145). While pregnancy-associated maternal death had received much attention, data concerning the impact of maternal infection on the brain development of children born to surviving mothers is scarce. As discussed above, experimental evidence strongly suggest that maternal infection can alter the developmental trajectory of fetal brain mainly by microglia. Few epidemiological studies have focused on pediatric patients in Africa (Gambia, Nigeria). In a cohort of 128 children in Gambia, a sizable fraction of these pediatric patients showed brain related delay such as learning difficulties (55%) and speech disorder (42%) (146). A similar set of studies in Nigeria show that children showed signs of epilepsy (60%), intellectual disability (7.2%) (147) and cerebral palsy (16.2%) likely due to early life events such as birth asphyxia and infection (148). These correlative studies strongly suggest that the prevalence of adult behavioral dysfunction and cognitive deficits in this African population is due, at least in part, to early life exposure to infectious pathogens.

Despite the prevalence of maternal infection, and its potential role in the emergence of such diseases as schizophrenia and autism spectrum disorder, few epidemiological and clinical trials have addressed these developmental diseases in Africa (149, 150). A recent study has shown that schizophrenic patients from South Africa (Xhosa ethnic group) carry damaging mutations in genes involved in synaptic function, such as receptors for glutamate and γ-amino-butyric acid (GABA) as well as postsynaptic proteins, scaffold proteins, and cell adhesion molecules (151). These mutations are comparable in nature to those observed in schizophrenic patients in Sweden (152). The mutation of these genes has been associated with intellectual disability, schizophrenia and autism spectrum disorders (153). It appears that the prevalence of schizophrenia is related to early life challenges such as childhood trauma (154). Similarly, maternal malaria has been associated with altered placental-fetal barrier by macrophage inflammatory mediators and complement factors (C5a), which can lead to altered fetal brain development (155).

The long term consequences of maternal infection on fetal brain development and function in sub-Saharan Africa has received little attention despite the overwhelming prevalence of infection during perinatal period. There is a need for studies that focus on the mechanistic link between perinatal infections and adult brain plasticity and function in Africa. These studies should take into consideration that subsaharan African mothers frequently experience multiple infections (parasitic, viral, bacterial) throughout pregnancy, which could be compounded with such factors as stress and malnutrition (156).

### Malnutrition and Neuroinflammation

Besides the lack or shortage of food, several sociocultural factors e.g. poverty, poor social infrastructure, food security, uncontrolled population explosion, land and crop degradation, and lack of access to health services, contribute to the rising levels of malnutrition in Africa (157, 158). Others factors include famine, limited knowledge about safe hygiene practices, pediatric environmental enteropathy (PEE), natural disasters as well as internal population displacements as a result of civil (religious or ethnic) unrest leading to children staying in unhygienic camps amongst others (159, 160). In particular, a strong link has been established between nutrition, inflammation and neurodevelopment from foetal life to adolescence on the continent (161). Malnutrition can be generally defined as the intake of insufficient, excess or disproportionate amount of energy and/or nutrients (162, 163). In all its manifestations, malnutrition presents as either (i) undernutrition, (ii) micronutrient imbalance, (iii) overnutrition and, (iv) diet-related non-communicable diseases (e.g. cardiovascular disease, stroke, diabetes etc.) (157, 163).

### Scope of the Problem

The statistics of numbers and people group affected by malnutrition creates a wide scope of problems in Africa. As a major global public health burden, the greatest concern is among infants, children, adolescents and women (particularly pregnant women) representing the most vulnerable category at greater risk of malnutrition (164–166). Globally in 2014, about 462 million adults worldwide were underweight, while 1.9 billion were either overweight or obese. An epidemiological study performed in 2016 showed that approximately about 155 million children under the age of 5 years were suffering from stunting, whereas 41 million were overweight or obese. In 2020, 40% of 149 million (59.6 million) stunted children under 5, 27% of 45 million (12.2 million) estimated to be wasted, and 24% out of 38.9 million estimated to be overweight or obese were from Africa (167; 163). The complicating fact however is that while there is a global decline in malnutrition, Africa has continued to record an increase in all forms of malnutrition, and for the most part, cases of undernutrition (168). This trend remains a serious concern as one of the leading cause of early child morbidity and mortality (157, 164).

The most common form of malnutrition recorded in developing countries most especially in Africa is undernutrition. Key indicators of undernutrition are wasting (low weight-for-height), stunting (low height-for-age) and underweight (low weight-for-age) (169, 170). Children under the age of five are the most severely affected of these vulnerable groups, with an estimated 45% of deaths attributed to undernutrition in this age group, mostly in low- and middle-income countries (170–172). Malnutrition is also responsible for significant abnormalities in physical and mental development with undernourished children usually having cognitive performance deficits and serious learning challenges (167, 173).

The continuous exposure of children and vulnerable groups to infectious agents under poor sanitary and unhygienic environment is of particular interest and has been shown to permanently weaken the immune systems and also cause a chronic inflammation of the intestine referred to as pediatric environmental enteropathy (PEE) in children (174–179). This gut disorder is as a result of both structural and functional changes in the intestinal mucosa characterized by intestinal villi atrophy, malabsorption, disruption of the intestinal gut barrier and an increased permeability (180, 181). This then makes it easier for microbes to translocate through the altered intestinal barrier. Over 75% of children in developing countries have been reported to affected by PEE (182).

### Maternal Malnutrition

Gressens et al. (183) noted a reduction on cortical astrogenesis in mice pups fed with low protein diet during the first fourteen days of gestation. Although the effect of malnutrition on the permeability of the blood-barrier (BBB) is not yet fully understood, alterations in astrocyte development might affect BBB formation (184, 185). Malnutrition during pregnancy causes a reduction on GFAP expression on rat hypothalamus (186) and hippocampus (187), and mice cerebellum (188). While several studies have reported a reduction on synaptic contacts after a period of malnutrition (189), recent data suggests that microglia dysfunction in their ability to respond to environmental stimuli during gestation and lactation affects synaptic plasticity *via* epigenetic regulatory mechanisms (67). In the adult brain, synaptic plasticity and basal neurotransmission has been found to be affected by certain soluble factors (e.g. BNDF) released by microglia (185).

In general, early‐life malnutrition in the form of overnutrition or undernutrition can have a lasting impact on astrocytes. Abbink et al. (190) posited that both overnutrition and undernutrition present with a very similar phenotype, specifically increased GFAP expression and glucose transporters. It is worthy of note that in the case of overnutrition, although energy levels remain high, a lack in nutrients might still occur. This could suggest that the observed changes are associated with alterations or shortages in circulating nutrients, changes in the metabolic profile, or just general energy imbalance, rather than it being a specific effect of either a lack or excess of energy. In a recent study conducted by Kogel et al. (191) on the effect of long-term semi-starvation on primary cortical rat astrocytes using an undernutrition model, authors provided morphological and genetic evidence for pro-inflammatory astrocyte subtype-induction suggesting that inflammatory processes are a relevant factor in undernutrition. This response is characterized by elevated pro-inflammatory cytokines and genes associated with starvation. Furthermore, a shift toward the pro-inflammatory A1-like phenotype and an altered morphology suggest an increased astrocytic reactivity.

### Crosstalk Between Malnutrition, Maternal Immune Activation and Neuroinflammation

Maternal immune activation (MIA) occurs when the measured levels of inflammatory markers in the dam exceeds normal range (192). It is usually a result of triggering of the maternal immune system by either infectious or non-infectious (malnutrition in this context) stimuli (193). This often leads to the release of inflammatory cytokines and immunologic alterations, and their transmission *via* innate placental immune activation to the developing foetus leading to adverse phenotypes particularly in the central nervous system (193–195).

There are strong emerging data from both animal and human studies that malnutrition-induced MIA results in foetal brain programming and modifications of their immune and metabolic genes through inflammatory and epigenetic mechanisms during critical periods of CNS astrocytes, microglial and immune system development (190, 194–197). Indeed, malnutrition during *in utero* and early life, notably due to undernutrition in the mothers, can affect the children’s growth, metabolism, immune function, brain, and cognitive development (198–200). Interestingly, neuroinflammation has recently been revealed as one of the key underlying mechanism responsible for deleterious consequences of diet-induced MIA on offspring neurodevelopment.

Microglial priming has been proposed as a major consequence of MIA, representing a vital connexion in a causal chain that leads to the wide spectrum of neuronal dysfunctions and behavioural phenotypes observed in the juvenile, adult or aged offspring. (201). In a study conducted by Ozaki et al. (202), authors observed maternal immune activation in mid-pregnancy led to an increase in IL-6 expression in embryonic microglia, but did not cause any marked changes in their morphologies either at E18 or after birth. However, they observed a sustained alteration in the microglial process motility pattern and deficits in behaviour when MIA was induced earlier (at E12).

These observations further strengthen the notion of the existence of a connecting link between maternal immune activation during pregnancy, and neuroinflammation and neurodevelopment disorders in the offspring. A significantly programmed imbalance in the expression of inflammatory mediators such as interleukin 6 (IL-6), IL-1 α, IL-10, tumor necrosis factor-α (TNFα), C-reactive protein or the complement system has been insinuated to play a role (118, 131, 134, 203–205).

**Figure 1:**
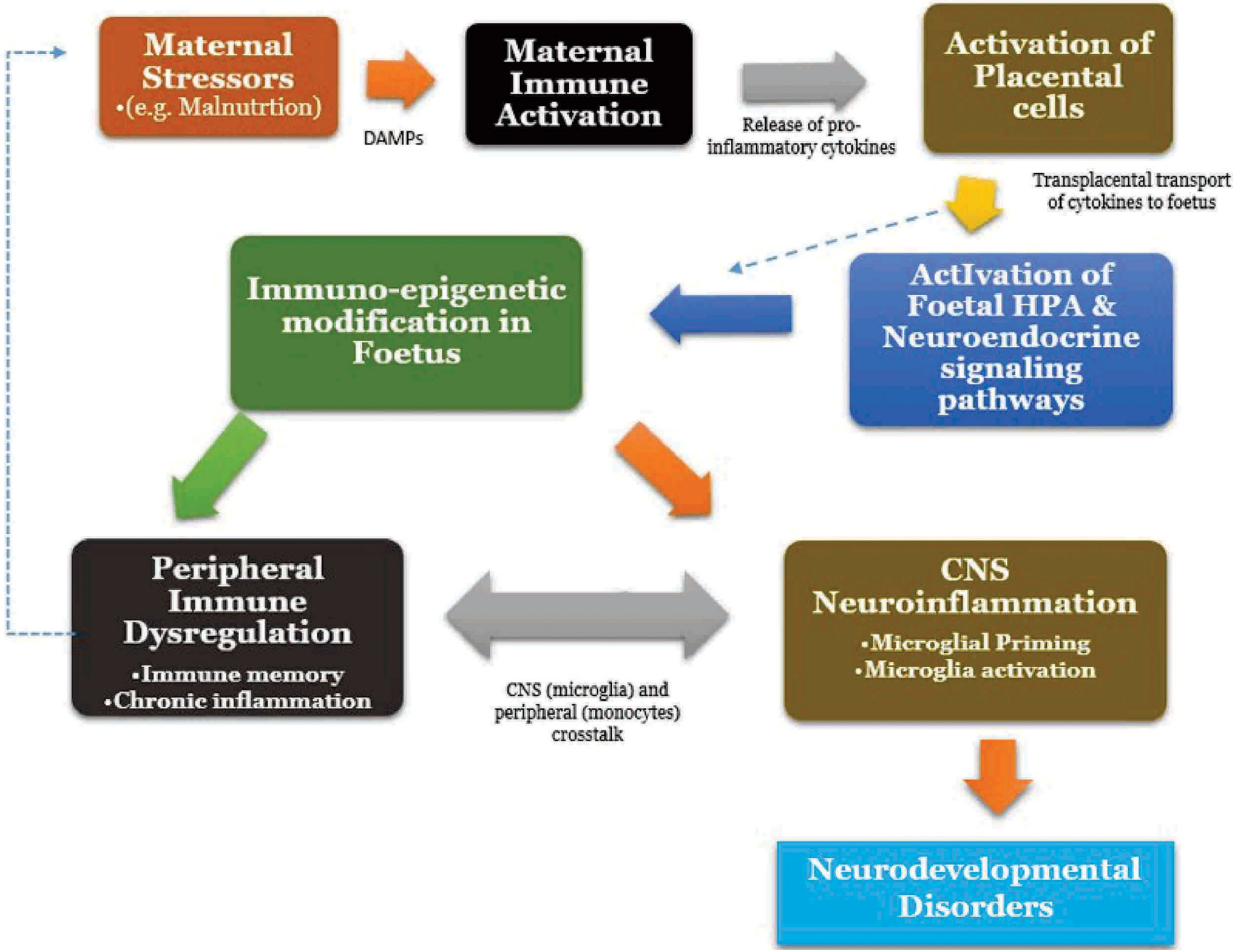
Malnutrition and Maternal immune activation, Neuroinflammation Crosstalk. HPA, Hypothalamic-Pituitary-Adrenal Axis.

Together, malnutrition-induced MIA induces the release of damage-associated molecular patterns (DAMPs), which then activates Toll-like receptors on maternal innate immune system and placental cells to produce pro-inflammatory cytokines (206–208). Following this, placental innate immune activation occurs and by means of passive transport as well as active placental production, cytokines across the placenta barrier with resultant interaction and activation of transplacental metabolic, hypothalamic–pituitary–adrenal (stress) and neuroendocrine signaling pathways (209). This consequently leads to foetal microglial priming, activation and neuroinflammation in the developing brain and also, the induction of immunological memory on the foetal microglia and the peripheral immune cells (194, 197). The resultant outcome is the occurrence of a dynamic crosstalk between the CNS immune cells (microglia) and peripheral immune cells (monocytes) (210). Second “hits” or wave of stress after birth (for instance by malnutrition) usually results in exaggerated responses and chronic inflammation in both the brain and periphery, manifesting as lifelong neurobehavioural deficits and may perpetuate a continuous cycle (194, 201, 211; **Figure 1**).

### The Vicious Tripartite Cycle of Malnutrition, Poverty and Neuroinflammation

**Figure 2:**
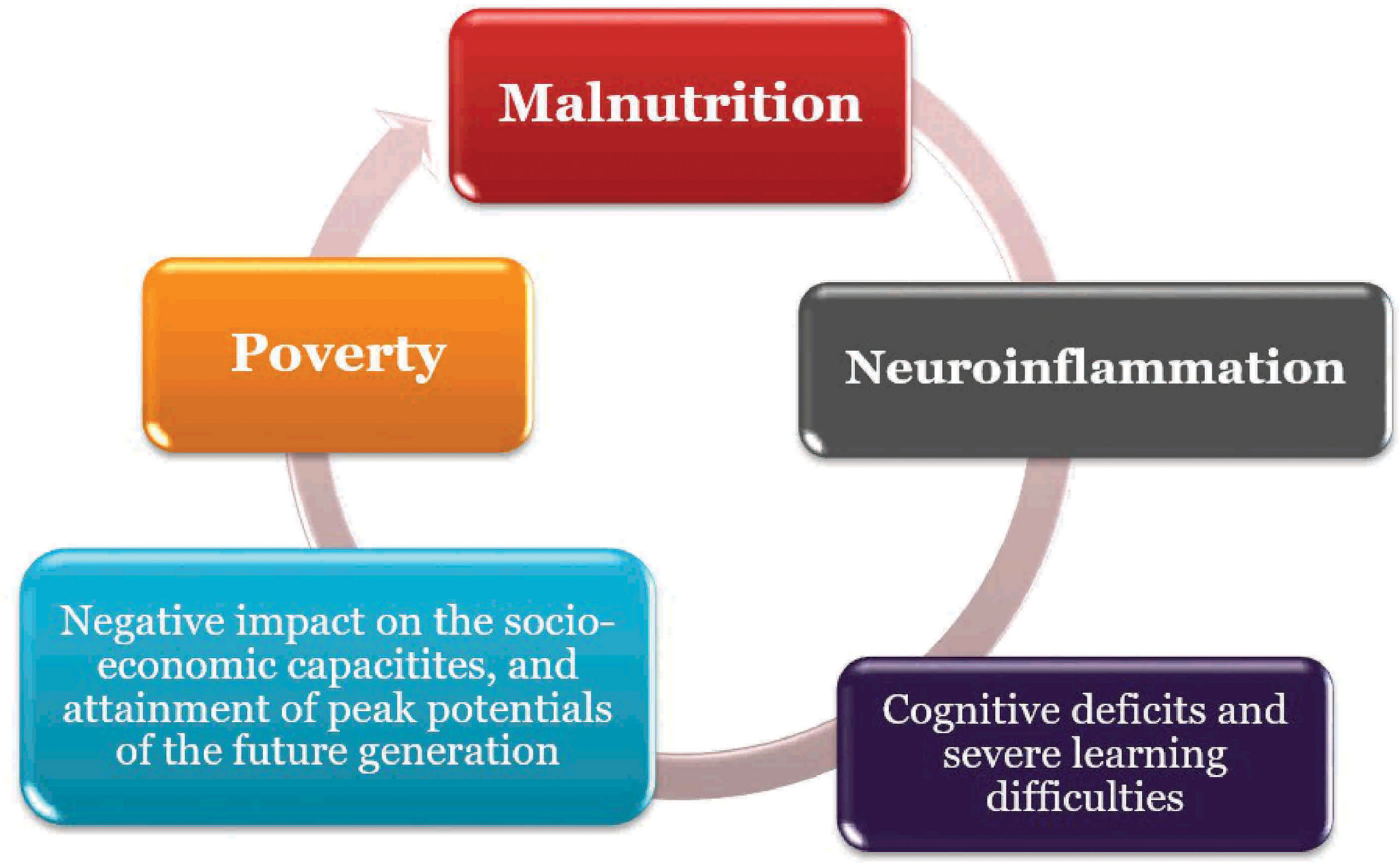
Vicious cycle of Malnutrition, Poverty and Neuroinflammation.

The relationships between nutrition, inflammation and neurodevelopment has been noted to be reciprocal; this further supports the concept of the vicious cycle posed by malnutrition (161, 163, 196). Poverty amplifies the risk of, and risks from, malnutrition. People who are poor are more likely to be affected by different forms of malnutrition. Also, malnutrition increases health care costs, reduces productivity, and slows economic growth, which can perpetuate a cycle of poverty and ill-health (161, 212; **Figure 2**). This portends significant risk for the African population viz neuroinflammation.

### Pollution-Induced Neuroinflammation in Africa

Africa is home to major stressors of the CNS that are known to alter the microglia-astrocyte physiology. This section reviews existing knowledge on glia interaction in the face of pollutants (metals, pesticides and contaminated air. Other than infectious diseases which are not fully addressed in the review, these pollutants pose as predisposing factors to CNS disorders in Africa, owing to the immense exponential rise in use and impact of chemicals in health, economic growth and sustainability especially in sectors of agriculture, mining, education and several other industrial processes. This has come with grave complications on communities of both users and non-users when exposed to these pollutants with bio-accumulation in the soil, water and in the air (213, 214). In a twist of tales, Africa is neither a major producer nor a consumer of chemicals in global terms, but has the highest levels of pollution because of non-existent or poorly implemented government environmental laws and waste disposal policies, poorly regulated mining sector, fossil fuel burning and wrong agriculture practices in the pharmaceutical, beverage, and food industries (215–217 and 214). From the lead (Pb) and cadmium (Cd) rich electronic dumpsite of Agbobloshie in Ghana, vanadium (V) rich crude oil and gas flares in Nigeria’s Niger-Delta, illegal mining in Congo and several other African countries, communities release multiple neurotoxic factors daily with the young and women being most vulnerable (218). Since early life stress produces altered neurobehavioural deficits in adult life (128). Many of these stress factors directly or indirectly easily cross the placenta, and the blood–brain barrier (which is not fully developed in in humans until about 6 months post-partum) and may lead to congenital malformations and risk of neonatal neurotoxicity (219–221). These pollutants exist in the form of several sulfides, sulfates, hydroxides, phosphates, silicates oxides, and organic compounds (222, 223) and may also cause acute or chronic effects on the CNS in the general population (216, 224, 225).

### Neuroinflammatory Mechanisms of Pollutants

Increasing evidence shows that astrocytes-microglia interplay may determine the phenotypic outcomes of the innate immune cells in disease conditions of the CNS. Glial activation can either aggravate tissue injury or promotes brain repair, most likely due to the nature of stress factors like the pollutant, dose and time course of exposure, and precise interplay of signals from the environment (226). Chemical pollutants include metals such as selenium, cadmium, arsenic, nickel, mercury, chromium, lead, zinc, and cobalt which are of paramount attention due to their potential role in toxicity when in trace amounts as well as other toxic pollutants such vanadium, tin oxide, copper etc. (227, 228). Other chemical pollutants include pesticides and air pollutants.

In metal pollution, microglia and astrocytes are known to express endogenous pattern recognition receptors (PRRs) in response to signals released by necrotic neurons or other pathologic products produced during disease including oxidized proteins and lipids (229), messenger ribonucleic acid (mRNA), fibronectin, hyaluronic acid, heat shock proteins, amyloid-beta, neuromelanin, and alpha-synuclein (230, 231). These are capable of responding to a variety of damage-associated molecular patterns (DAMPs) and in turn activate inflammation and neurodegeneration promoting molecular signaling events (232). The production of inflammatory mediators is further increased by activated glia, leading to a feed-forward cycle of inflammation and further release of neurotoxic mediators of tissue injury. The activated glia release diverse inflammatory factors including cytokines, chemokines, reactive oxygen species (ROS), and nitric oxide (NO) that are toxic to neurons (233). Cytokines such as tumor necrosis factor-alpha (TNFα) and interleukin-6 (IL-6) are often upregulated very quickly in activated glial cells and can directly amplify inflammation through recruitment of both innate and adaptive immune cells, leading to neuronal apoptosis (231).

When exposed to pollutants, microglia and astrocytes typically increase the production and release of inflammatory cytokines which enhance (ROS) generation, impede antioxidant activity, and result in neuronal injury or neuronal loss in the brain or other parts of the CNS (234). While the precise mechanisms are not yet fully understood, microglia have been shown as the first line of action and they respond in a dose-dependent fashion, while astrocytes are known to accumulate more toxic elements and express cytokines much later. Exposure to metals such Lead (Pb), Methyl mercury (MeHg), Vanadium (V), Tin Oxide (TO) results in gliosis by activating Toll-like receptor 4 (TLR4) -myeloid differentiation primary response 88 (MyD88) -nuclear factor (NF)-κB signaling cascade, increasing receptor phosphorylation and the activation of Mitogen-activated protein kinase (MAPK) cascades with subsequent initiation of signal transduction some of which are responsible for the production of pro-inflammatory cytokines (218, 234–236). This exposure is associated with upregulated activation of nuclear factor erythroid 2–related factor 2 (Nrf2) which acts against electrophiles and oxidants in the detoxification of ROS to maintain homeostasis (237). When exposed to ROS, Nrf2 acts by separating from the cytoplasmic repressor protein - Kelch-like ECH-associated protein 1 (Keap1), transferring to the nucleus, and activating the expression of antioxidant response elements (ARE)-dependent genes, including the phase II detoxifying/antioxidant enzyme HO-1 and NQO1 (238). The activation of the apoptotic caspase-3 pathway, which results in neuronal damage neurons is also suggested (239). This mechanism induces primary microglial toxicity and may be the pathological basis of metal pollution induced neurological dysfunction.

### Mercury

MeHg pollution inhibits the astrocytic uptake of cysteine; an essential precursor for glutathione (GSH) synthesis. Implying that MeHg pollution induces neuronal oxidative damage (240). Part of the inhibitory mechanisms of MeHg include astrocytic glutamate uptake inhibition and glutamate efflux (241). This results in excessive glutamate in the synaptic cleft and, consequently leads to neuronal excitotoxicity. *In vivo*, mercury has been shown to induce microglial production and secretion of lysosomal proteases, leading to neuronal toxicity while astrocytes, when co-cultured with neurons, increase neuronal resistance to the damaging effect of MeHg (242, 243). Astrocytes and microglia therefore mediate protective effects against MeHg-induced neuronal toxicity. Microglia increase interleukin-6 (IL-6) production and release (244).

In organotins however, *In-vitro* studies have shown increased expression of IL-1β, tumor necrosis factor (TNF-α), IL-6, and nitric oxide synthase (iNOS) in the cultured astrocytes and microglia (245, 246).

### Manganese (Mn)

Molecular mechanisms involved in Mn-induced neurotoxicity involve direct damage to the substantia nigra, globus pallidus, basal ganglia, striatum, and various other cellular components of the nervous system. Mn accumulates in the mitochondria of various cellular components in the brain, causing F_0_/F_1_ synthase and succinate dehydrogenase abnormality, leading to reduced ATP production (247). Diminishing ATP levels increase intracellular calcium levels and induce severe oxidative stress, forming ROS. Manganese was also shown to oxidize dopamine into reactive quinone species and disruption of antioxidant enzymes *via* binding to their thiol and hydroxyl groups (248). Glia activation has however been shown to occur in astrocytes and microglia in manganism (249) as it potentiates the effects of LPS and cytokines on activation of both microglia and astrocytes leading to increased production of TNFα, IL-1β, ROS, and NOS2 expression that can cause neuronal injury (250).

Manganese activates NF-κB and mitogen-activated protein kinase (MAPK) in microglia resulting in inflammatory gene expression and production of inflammatory mediators (251). The inflammatory effects are tightly regulated both at the level of IKK activation as well as by nuclear proteins that modulate transcriptional activity of inflammatory genes- NR4A1 (Nurr1) (252). Microglia then release neuroinflammatory mediators and pro-inflammatory cytokines, as well as reactive oxygen and nitrogen species (ROS and RNS), all of which can act on astrocytes to amplify inflammatory responses in the CNS (250).

In astrocytes, higher levels of accumulation of Mn occur than in neurons. This makes them target cells for transport of Mn into the brain as well as for initiating inflammatory signaling during neuronal stress and injury. Since astrocytes are a heterogeneous population of cells with different morphological and physiological characteristics depending on their location with the brain (253), they invariably serve as the major homeostatic regulator and storage site for Mn in the brain and a prominent contributor to Mn-stimulated nitric oxide (NO) production through NOS2 (254, 255). The regulation of astrocyte activation is under the control of many factors including cytokines IL-6, IFNγ, tumor necrosis factor-alpha (TNFα), toll-like receptor activators, neurotransmitters, ATP, reactive oxygen species, hypoxia, glucose deprivation, ammonia, and protein aggregates (256). Frequently, these activators are by-products of already injured neurons or factors released by activated microglia which indicate that astrocyte activation is often later in disease progression (257).

Cell culture models of glia cross talk in managanism indicate that removal of microglia or use of antioxidants has shown to reduce neuronal loss indicating microglial activation may serve as a critical step in mediating neuronal injury during Mn exposure and that microglia also likely directly promote activation of astrocytes that then amplify neuronal damage (258). However, astrogliosis is often more persistent than microgliosis and is believed to be important in amplifying inflammatory processes and thereby inducing greater damage (259).

### Pesticides

Over 45% of neurotoxic chemicals are pesticides. Exposure to toxic doses of these chemicals activates the CNS immune system by reducing Nrf2 activation, activating the NF-kB pathway, or the opening of voltage gated calcium channels in neurons. These lead to increased oxidative stress, neuroinflammation, neuronal apoptosis, activation of p38MAPks, nucleotide-binding domain, leucine-rich repeat (NLR) family pyrin domain containing 3 (NLRP3) inflammasome, and reduced serotonin. Examples include the organophosphates which primarily cause accumulation of acetylcholine at cholinergic synapses, resulting in muscarinic and nicotinic receptor over-stimulation leading to oxidative stress, lipid peroxidation (260). Organophosphates can also alter the cyclic-AMP-protein kinase A signaling pathway of which affects the expression and function of several nuclear transcription factors such as c-fos, p53, AP-1, Sp1 and CREB (Ca2+/cAMP response element binding protein) involved in the switch from proliferation to differentiation of neural cells (260).

Dieldrin is an organochlorine extensively used as pesticides for corn, cotton, and citrus crops has been reported to induce severe alteration in the function of dopaminergic neurons and GABA_A_ receptor (261) with evidence of significant oxidative stress, mitochondrial dysfunctions, and generation of pro-apoptotic proteins such as caspase-3 and Bcl-2 in the dopaminergic neurons (262). Endosulfan is an off-patent organochlorine insecticide and acaricide. It has been used globally as a pesticide since the 1950s to control a variety of insects including whiteflies, aphids, leafhoppers, Colorado potato beetles, and cabbage worms applied extensively to coffee, tea, and cotton crops, among others (263). It induces severe oxidative stress, induces the expression of pro-apoptotic proteins and inflammatory cytokines, and activation of glial cells (264). Pyrethroids are synthetic insecticides, which are used for the controlling insect pests in agriculture, public health, and animal health. They mediate prolongation of the kinetics of voltage-gated sodium channels, which are responsible for generation of the inward sodium current that produces the action potential in excitable cells leading to a hyperexcitable state, damage BBB and cause induction of severe endoplasmic reticulum stress, neuronal apoptosis, microglial activation, and neuroinflammation (265, 266).

Rotenone and pyridaben are two mitochondrial complex I inhibitors and are highly lipophilic. They easily cross BBB and produce ROS, Ca^2+^-mediated hyperexcitation, nuclear translocation of NF-kB, activation of p38 MAPKS, the formation of NLRP3 inflammasome, and mitochondrial dysfunctions (267–270).

### Traffic Related Air Pollutants

TRAP exposure induces oxidative stress products, such as malondialdehydes (MDA), thiobarbituric acid reactive substances (TBARs) as well as ROS such as H_2_O_2._ (Nrf2), superoxide dismutase (SOD), glutathione (GSH), heme oxygenase 1 (HO-1), and catalase (CAT) are commonly elevated in the central nervous system, indicating need for detoxification. This induction activates glia response with astrocytic activation usually occurring either concomitantly with, or immediately after microglia stimulation, thus contributing to the release of oxidant species and pro-inflammatory cytokines (222, 271, 272).

Diesel Exhaust (DE): has been shown to induce oxidative stress, to activate microglia and to enhance levels of several pro-inflammatory cytokines (IL-1a, IL-1b, IL-3, IL-6, TNF-α) in the olfactory bulb and the hippocampus and microglia activation resulting in decreased adult neurogenesis in the hippocampal subgranular zone (SGZ) and the subventricular zone (SVZ) [Reviewed in (222, 273)].

## Conclusion

In summary, multiple factors not limited to those discussed in this review may modulate neuroinflammation within the African context. These stressors assault the CNS through several cellular and molecular pathways to modulate neuroinflammatory responses that can be traced back to early development, with possible persistence into adult life and risk of mortality (274, 275). The most usually implicated pathways have oxidative stress, cerebral vascular damage, neurodegeneration and infiltrating systemic inflammation or nanoparticles as major route to damage. Microglia and astroglia respond to these stressors *via* multiple mechanisms that are still a subject of intense investigations. With the continent being home to over 1.3 billion people with myriads of stressors, the increasing burden of neurological disorders may be a ticking time bomb for neurological disorders (276). Therefore, further research in collaboration with Africans on epidemiological and mechanistic studies into the association of stressors and neuroinflammation will go a long way in understanding pathways that may be beneficial in treating or managing cases. Such studies will help to determine the neurological disease burden and to what extent these stressors contribute to neurological disease progression, co-morbidities with other neurodegenerative diseases and mortalities, by looking at the genetic and molecular adaptations and or vulnerabilities that exist in the African space compare to their Western cohorts.

## Author Contributions

MO, AM, CF and FQ contributed to concept note development. MO wrote the abstract and conclusion, AM wrote section on microglia, CF wrote on astrocytes, FQ contributed the astrocyte and microglia cross talk section, OM wrote on malnutrition, MO and JO wrote on environmental pollutants, JO supervised all contributions of MO. OM worked on references with the assistance of MO. All authors contributed equally to manuscript proof reading and editing.

## Funding

CF’s research is supported by Italian Ministry for Health (Ricerca Corrente and RF-2018-12367731), FISM (Fondazione Italiana Sclerosi Multipla, grant number 2016/R/14) and cofinanced with the 5 per mille public funding. AM was supported by research grants from Kuwait University, Research Sector (YM04/21 and YM11/17).

## Conflict of Interest

The authors declare that the research was conducted in the absence of any commercial or financial relationships that could be construed as a potential conflict of interest.

## Publisher’s Note

All claims expressed in this article are solely those of the authors and do not necessarily represent those of their affiliated organizations, or those of the publisher, the editors and the reviewers. Any product that may be evaluated in this article, or claim that may be made by its manufacturer, is not guaranteed or endorsed by the publisher.
